# Vaccine hesitancy and refusal: perspectives of midwives working in family health centers – a descriptive cross-sectional study

**DOI:** 10.1590/1980-220X-REEUSP-2025-0469en

**Published:** 2026-09-07

**Authors:** Şeyma Çatalgöl, Eda Can

**Affiliations:** 1 Uşak University, Faculty of Health Sciences, Department of Nursing, Uşak, Türkiye.; 2 İzmir City Hospital, İzmir, Türkiye.

**Keywords:** Midwives, Vaccination, Vaccine Refusal, Vaccination Hesitancy, Tocologia, Vacinação, Recusa de Vacinação, Hesitação Vacinal

## Abstract

**Objective:**

This study aims to determine the views of midwives working in family health centers regarding vaccine hesitancy and refusal, as well as the solution strategies they propose for this issue.

**Method:**

This descriptive cross-sectional study was conducted with 81 midwives working in family health centers in the province of Uşak, Türkiye, between March 24 and May 18, 2021. Data were collected via Google Forms using a structured questionnaire developed based on the literature. The data were analyzed using descriptive statistics, including frequencies and percentages.

**Results:**

According to midwives’ reports, the most frequently cited factors contributing to vaccine hesitancy and refusal include exposure to anti-vaccine websites (90.1%), concerns about vaccine ingredients (70.4%), the influence attributed to community leaders (66.7%), and religious beliefs (65.4%). Other reported factors include past vaccination experiences (56.8%), distrust of vaccine manufacturers (44.4%), and distrust of healthcare workers and the healthcare system (38.3%). An analysis of vaccine information sources revealed that patients most frequently used the internet (37.0%), their social environment (30.9%), healthcare institutions (24.7%), and television (7.4%). 65.4% of midwives reported encountering anti-vaccine individuals. The most frequently recommended strategies include: presenting television content supporting vaccination (70.4%), broadcasting public service announcements via television and the internet (67.9%), and providing information about vaccines through healthcare workers (58.0%).

**Conclusion:**

Midwives’ field observations suggest that vaccine hesitancy and refusal may be associated with digital misinformation, trust-related concerns, and sociocultural factors. Strengthening education initiatives led by healthcare workers and media-based information strategies could potentially contribute to increasing vaccine acceptance.

## RESUMO

**Objetivo:**

Este estudo tem como objetivo determinar as opiniões das parteiras que trabalham em centros de saúde da família sobre a hesitação e a recusa em relação à vacinação, bem como as estratégias de solução que propõem para essa questão.

**Método:**

Este estudo descritivo transversal foi realizado com 81 parteiras que trabalham em centros de saúde da família na província de Uşak, na Turquia, entre 24 de março e 18 de maio de 2021. Os dados foram coletados por meio do Google Forms, utilizando um questionário estruturado desenvolvido com base na literatura. Os dados foram analisados utilizando estatística descritiva, incluindo frequências e percentuais.

**Resultados:**

De acordo com os relatos das parteiras, os fatores mais citados que contribuem para a hesitação e a recusa à vacinação incluem a exposição a sites antivacinas (90,1%), preocupações com os ingredientes das vacinas (70,4%), a influência atribuída aos líderes comunitários (66,7%) e crenças religiosas (65,4%). Outros fatores relatados incluem experiências anteriores com vacinação (56,8%), desconfiança nos fabricantes de vacinas (44,4%) e desconfiança nos profissionais de saúde e no sistema de saúde (38,3%). Uma análise das fontes de informação sobre vacinas revelou que as pacientes utilizavam com maior frequência a internet (37,0%), seus círculos sociais (30,9%), instituições de saúde (24,7%) e a televisão (7,4%) enquanto 65,4% das parteiras relataram ter encontrado indivíduos antivacinas. As estratégias mais frequentemente recomendadas incluem: apresentar conteúdo televisivo que apoie a vacinação (70,4%), veicular anúncios de utilidade pública pela televisão e pela internet (67,9%) e fornecer informações sobre vacinas por meio de profissionais de saúde (58,0%).

**Conclusão:**

De acordo com as observações de campo das parteiras, a hesitação e a recusa em relação à vacinação são vistas como um fenômeno multidimensional que envolve uma combinação de desinformação digital, questões relacionadas à confiança e fatores socioculturais. O fortalecimento de iniciativas educacionais lideradas por profissionais de saúde e estratégias de informação baseadas na mídia poderiam contribuir para aumentar a aceitação da vacinação.

## INTRODUCTION

Vaccine hesitancy and vaccine refusal are defined as individuals delaying or refusing vaccination despite the availability of vaccination services. Although vaccination is one of the most cost-effective public health interventions for preventing infectious diseases, vaccine hesitancy and refusal remains a significant global health issue today. The World Health Organization (WHO) identified vaccine hesitancy as one of the top ten threats to global health in 2019^(1)^. According to current data, approximately 14.3 million children worldwide remained unvaccinated in 2024; these children are referred to as “zero dose children.” During the same period, the global vaccination coverage rate for the third dose of the DTP3 vaccine which provides protection against diphtheria, tetanus, and pertussis was reported to be 85%. These results indicate that significant gaps in vaccination services still exist^(2)^.

Vaccine hesitancy is defined as being associated with numerous factors, such as concerns about vaccine safety, difficulties in accessing vaccines, and individual neglect. In this process, healthcare workers are regarded as the most reliable source of information that can influence individuals’ decisions regarding vaccination and play a critical role in providing effective counseling^(3)^. Healthcare workers’ abilities to build trust, clarify conflicting information, and provide evidence-based recommendations are among the key factors in reducing vaccine hesitancy and refusal^(4,5)^.

Among healthcare workers, midwives hold a unique and critical position, particularly in primary care, due to the continuous and close contact they establish with women throughout pregnancy, childbirth, and the postpartum period. Midwives assume the responsibility of providing information and guidance regarding both maternal and childhood vaccinations. Given the increasing emphasis on vaccination during pregnancy and the need for parents to be informed about vaccines during the prenatal period, midwives play a key role in providing vaccination counseling and administering vaccination services^(6)^.

Managing conversations with individuals who express vaccine hesitancy or refusal, addressing their concerns, and providing reliable information are among midwives’ fundamental professional and ethical responsibilities. Fulfilling these responsibilities through a non-judgmental, respectful, and woman-centered approach enables midwives to make significant contributions to processes aimed at increasing vaccine acceptance and supporting public health^(7)^.

However, midwives’ own levels of confidence in vaccines and potential hesitations can influence their tendency to recommend vaccines to individuals. A strong relationship has been demonstrated between healthcare workers’ confidence in vaccines and the public’s attitude toward vaccination^(8)^. Furthermore, it has been reported that midwives may express greater concerns regarding vaccine safety compared to other healthcare workers and may be more hesitant to recommend vaccines, particularly during pregnancy. This underscores the importance of strengthening midwives’ knowledge and confidence regarding vaccines^(9)^.

On the other hand, the widespread use of digital platforms significantly influences the flow of information regarding vaccines. When healthcare workers, particularly midwives, share inaccurate or anti-vaccine information in digital environments, it can undermine public trust in health messages. Therefore, providing scientific and reliable information both in clinical practice and on digital platforms is an integral part of midwives’ professional responsibilities^(10)^.

Despite midwives’ critical role in vaccination services, there is a limited number of studies that comprehensively address the real life clinical experiences of midwives particularly those working in primary care regarding vaccine hesitancy and refusal, as well as the solution strategies they have developed to address this situation. Current research primarily focuses on specific interventions, and comprehensive evaluations based on field practices remain insufficient^(7,11)^.

In this context, studies that reflect field practices and are grounded in midwives’ experiences are important for contributing to the development of approaches to address vaccine hesitancy. Accordingly, this study aims to identify the views of midwives working in family health centers regarding vaccine hesitancy and refusal, as well as the solution strategies they propose for this situation.

## METHODS

### Study Design

This study was designed as a descriptive cross-sectional study. Due to its descriptive nature, no analyses of relationships or causality between variables were conducted; the data were evaluated using only descriptive statistics.

### Study Setting

The research was conducted at family health centers in Uşak Province, located in Türkiye’s Inner Aegean Region. In Türkiye, primary health care services are largely provided through family health centers, and midwives play an active role in preventive health services such as prenatal care, postpartum care, administration of childhood vaccinations, and routine family health services. Therefore, midwives are key healthcare professionals who directly encounter vaccine hesitancy and refusal.

The family health centers where the participants work have between two and six physicians and midwives on staff. Each physician works with one midwife, and each team serves a population of approximately 4,000 people. Vaccination programs in Türkiye are carried out in a coordinated manner; while the postnatal hepatitis B vaccine is administered to newborns in the hospital, other childhood vaccines, tetanus vaccines for pregnant women, and other routine vaccines are administered at family health centers. This context provides a work environment that could directly influence midwives’ observations regarding vaccine hesitancy and refusal.

### Inclusion and Exclusion Criteria

The study included all midwives aged 18–65 working at family health centers in the province where the research was conducted who provided informed consent electronically. Midwives who did not provide electronic consent were excluded from the study.

### Participants and Sampling

The study was conducted between March 24 and May 18, 2021. The study population consisted of a total of 88 midwives working at 23 family health centers in the relevant province. Given the relatively small and manageable size of the population, it was aimed to reach the entire population. This approach was intended to minimize sampling error and to enhance the representativeness of the data for midwives working in family health centers across the province. Of the 88 midwives comprising the study population, 81 voluntarily participated in the study; seven midwives chose not to participate. The voluntary participation rate was 92%, which enhanced the representativeness of the data. Reminder emails were also sent to encourage participation.

### Data Collection Tool

Data were collected using a questionnaire developed in accordance with the literature^(4,5,6,7,8,9,10,11,12,13)^, consisting of 27 multiple-choice questions. The survey consisted of four sections: (1) descriptive characteristics of the midwives; (2) perceptions of vaccine refusal/hesitancy; (3) perceptions regarding vaccine hesitancy and refusal, including beliefs, information sources, and professional exposure; and (4) proposed solutions for preventing vaccine hesitancy/refusal.

### Validity and Reliability

The content validity of the questionnaire was assessed by three academics specializing in midwifery. The experts were asked to review each item based on the criteria of coverage (the extent to which the item covers the relevant topic), clarity (whether the wording is clear and understandable), and appropriateness (the item’s alignment with the research objective). Based on the experts’ feedback, some items underwent wording changes and language revisions.

A pilot test of the survey was conducted with 15 midwives working in a different province, resulting in minor corrections to wording and word choice in three items; no changes were required for the remaining items. The midwives who participated in the pilot test were not included in the main study.

Since the questionnaire consisted of multiple thematic sections rather than a single-dimensional scale, an overall Cronbach’s alpha coefficient was calculated as a general indicator of internal consistency (α = 0.87).

### Data Collection

Due to the COVID-19 pandemic and the need to limit face-to-face contact, data were collected online. The questionnaire was prepared using Google Forms and distributed to participants via email. Reminder messages were sent to increase participation.

### Ethical Approval and Informed Consent Statements

The study was approved by the Uşak University Clinical Research Ethics Committee (Approval Date: 17 March 2021, Number: 69-69-06) and conducted in accordance with the ethical guidelines established in the Declaration of Helsinki. Permission was also obtained from the Uşak Provincial Health Directorate, where the primary healthcare services involved in the study are affiliated (Date: 3 March 2021, Number: E-49998565-604.02.03). Written informed consent was obtained from midwives who agreed to participate in the study. All participants were informed about the purpose and design of the study.

### Statistical Analysis

Data were analyzed using the SPSS 22.0 software package. Categorical variables were presented as frequency (n) and percentage (%). Due to the descriptive nature of the study, no statistical relationship or causality analysis was conducted between variables.

## RESULTS

### Results Regarding the Demographic Characteristics of Midwives

A total of 81 midwives participated in the study. The sociodemographic characteristics of the participants are presented in Table 1. It was determined that 44.4% of the participants were in the 35–44 age group and 85.2% were married. In terms of professional experience, the most common duration was 6–10 years (28.4%) and 0–5 years (24.7%). It was found that the majority of participants had one (34.5%) or two (38.3%) children (Table 1).

**Table 1:** Descriptive characteristics of the midwives (N = 81) – Uşak, TR, Türkiye, 2021.

	n = 81	(%)
**Age**		
18–24	12	14.8
25–34	26	32.1
35–44	36	44.4
45–64	7	8.6
**Marital Status**		
Married	69	85.2
Single	12	14.8
**Professional Experience (year)**		
0–5	20	24.7
6–10	23	28.4
11–15	11	13.6
16–20	11	13.6
21–25	10	12.3
26–30	4	4.9
≥31	2	2.5
**Number of Children**		
0	10	12.3
1	28	34.5
2	31	38.3
3	11	13.6
≥4	1	1.2

### Midwives’ Reports on Vaccine Hesitancy and Refusal

Midwives’ reports on vaccine hesitancy and refusal are presented in Table 2. The most frequently reported factors include exposure to anti-vaccine websites (90.1%), concerns about vaccine ingredients (70.4%), the influence attributed to community leaders (66.7%), and religious beliefs (65.4%). These are followed by past vaccination experiences (56.8%), distrust of vaccine manufacturers (44.4%), and distrust of healthcare workers and the healthcare system (38.3%) (Table 2).

**Table 2:** Midwives’ perceptions on vaccine refusal/hesitancy (N = 81) – Uşak, TR, Türkiye, 2021.

	Yes n (%)	No n (%)	Partially n (%)
Religious beliefs	53 (65.4)	10 (12.3)	18 (22.2)
Anti-vaccine websites	73 (90.1)	3 (3.7)	5 (6.2)
The influence attributed to community leaders	54 (66.7)	10 (12.3)	17 (21)
Transportation problems to healthcare facilities	22 (27.2)	42 (51.9)	17 (21)
Past vaccination experiences	46 (56.8)	20 (24.7)	15 (18.5)
Distrust of healthcare workers and the health system	31 (38.3)	26 (32.1)	24 (29.6)
Distrust of vaccine manufacturers	36 (44.4)	20 (24.7)	25 (30.9)
Concerns about vaccine ingredients	57 (70.4)	16 (19.8)	8 (9.9)

Table 3 presents midwives’ perceptions regarding individual beliefs, patients’ sources of information, and professional experience in the context of vaccine hesitancy/refusal. Among the most frequently reported concerns regarding individual beliefs were concerns that vaccines may have unknown long-term effects (48.1%), may cause autism or autoimmune diseases (37.0%), and have no benefits (32.1%). These were followed by perceptions that vaccine manufacturers are of foreign origin (24.7%), beliefs that organic foods can replace vaccines (22.2%), and that vaccines may cause infertility (6.2%) (Table 3).

**Table 3:** Midwives’ perceptions regarding vaccine hesitancy and refusal: beliefs, information sources, and professional exposure (N = 81) – Uşak, TR, Türkiye, 2021.

Item	n (%)
Belief that organic foods replace vaccines	18 (22.2)
Belief that vaccines have no benefit	26 (32.1)
Belief that vaccines cause autism/autoimmune disease	30 (37)
Belief that vaccines may have unknown long-term effects	39 (48.1)
Foreign origin of vaccine manufacturers	20 (24.7)
Belief that vaccines may cause infertility	5 (6.2)
Midwives’ perceptions of patients’ sources of vaccine information*	
Television	6 (7.4)
Internet	30 (37)
Social environment (friends/neighbors)	25 (30.9)
Healthcare institutions	20 (24.7)
**Exposure to vaccine hesitancy/refusal cases**	
Encountered	53 (65.4)
Not encountered	28 (34.6)

*Categories marked Multiple responses possible allow respondents to select more than one option. Percentages are standardized to one decimal place.

In terms of information sources, it was determined that patients most frequently use the internet (37.0%), followed by social environment (30.9%), healthcare institutions (24.7%), and television (7.4%). 65.4% of midwives reported encountering anti-vaccine individuals (Table 3).

### Suggested Solutions for Midwives’ Vaccine Hesitancy/Refusal

Suggestions for reducing vaccine hesitancy and refusal among midwives are presented in Table 4. Upon reviewing the results, it is evident that the suggestions largely focus on media-based information campaigns and educational activities conducted through healthcare workers. The most frequently recommended approach was including pro-vaccination content in television programs (70.4%). The next more frequent recommendation was the regular broadcast of public service announcements on television and the internet (67.9%) and providing public education through healthcare workers about vaccine ingredients, side effects, and the risks of non-vaccination (58.0%). Other recommended strategies included providing information about the benefits of vaccines during civil society activities (55.6%), regular vaccination campaigns (49.4%), educating people about the importance of vaccination in prenatal classes, women’s associations, and public education centers (43.2%), information on herd immunity by healthcare professionals (40.7%), and mandatory vaccination policies (40.7%) (Table 4).

**Table 4:** Midwives’ proposed solutions for preventing vaccine hesitancy/refusal (N = 81) – Uşak, TR, Türkiye, 2021.

Proposed solutions by midwives	n	%
Including pro-vaccination content in television programs	57	70.4
Providing information about the benefits of vaccines during civil society activities	45	55.6
Public service announcements on TV and internet	55	67.9
Regular vaccination campaigns	40	49.4
Providing public education through healthcare workers about vaccine ingredients, side effects, and the risks of non-vaccination	47	58
Educating people about the importance of vaccination in prenatal classes, women's associations, and public education centers	35	43.2
Information on herd immunity by healthcare professionals	33	40.7
Mandatory vaccination policy	33	40.7

## DISCUSSION

This study demonstrates that vaccine hesitancy and refusal exhibit a multidimensional structure, based on midwives’ field observations. The findings suggest that attitudes toward vaccination are not limited solely to individual knowledge levels; rather, they emerge within a complex framework that must be considered in conjunction with digital information environments, cultural belief systems, social interaction spaces, and institutional trust dynamics.

Participants’ statements reveal that digital environments, and particularly anti-vaccine content, are becoming increasingly influential in health communication processes. The widespread use of online platforms for accessing health information can facilitate the dissemination of unverified content to wider audiences^(12)^. Restricting anti-vaccine content on social media and other digital platforms can be considered an important step toward strengthening vaccine confidence^(13)^.

Concerns about vaccine components and perceptions of safety constitute another prominent theme in participants’ views. The literature indicates that similar concerns are widespread across different populations and that misconceptions regarding the long-term effects of vaccines are particularly influential^(14)^. The findings are consistent with the existing literature^(5,15)^, indicating that risk perception and trust issues lie at the core of vaccine hesitancy. It is assessed that transparent and evidence based communication strategies could play a critical role in reducing these perceptions^(16)^.

Social and cultural factors, particularly religious beliefs and the influence of community leaders, constitute another notable aspect in the participants’ statements. The literature also indicates that religious beliefs can be a determining factor in vaccination decision-making processes^(17)^. This finding demonstrates that health behaviors are shaped not only by individual decision-making processes but also by cultural and faith-based norms. Therefore, it is believed that awareness campaigns conducted through religious authorities could help reduce information gaps^(18)^.

Individual experiences and negative experiences related to past vaccination processes also emerge as a significant theme. The literature indicates that previous experiences of side effects are associated with vaccine hesitancy^(19)^. Strengthening vaccine safety and transparently monitoring side effects could help alleviate these concerns^(20)^.

Distrust toward vaccine manufacturers is another key element highlighted in participants’ statements. The literature indicates that individuals’ perception of vaccine manufacturers as profit-driven can increase vaccine hesitancy^(14)^. This suggests that increasing transparency in vaccine development and licensing processes could be crucial for building trust^(3)^.

Trust in healthcare workers and the healthcare system is another key theme that emerged in the participants’ views. The literature indicates that healthcare professionals’ communication skills and their ability to build trust-based relationships play a critical role in increasing vaccine acceptance^(21)^. This finding highlights the need to strengthen not only the transmission of information but also trust-based communication in interventions.

Midwives’ field observations point to the diversity of individuals’ information sources. The internet, social circles, healthcare institutions, and traditional media have been reported as effective information sources to varying degrees. The literature emphasizes that digital information environments form a complex ecosystem that shapes health decisions^(22)^. Therefore, strengthening the position of healthcare professionals as reliable sources of information is crucial.

According to participants, the key factors contributing to vaccine hesitancy and refusal include concerns about long-term effects, associations with autism and autoimmune diseases, a perception of ineffectiveness, and distrust of manufacturers. Additionally, misconceptions such as the belief that organic food is protective, as well as that vaccines carry the risk of infertility have been reported. The literature indicates that such misinformation is particularly disseminated through digital platforms and erodes vaccine confidence^(23,24,25)^. Therefore, enhancing health literacy and developing targeted communication strategies are of great importance^(26)^.

In the study, a significant proportion of midwives reported encountering cases of vaccine hesitancy and refusal. This finding is consistent with the literature indicating that vaccine hesitancy and refusal are prevalent in various populations^(27,28)^. This situation underscores the importance of improving health literacy and disseminating reliable information through healthcare professionals.

Among the midwives’ recommendations, media-based awareness campaigns and information strategies delivered by healthcare workers stand out. In particular, public service announcements to be broadcast on television and the internet are considered a key area for intervention. This finding is consistent with studies highlighting the media’s influence on vaccine acceptance^(29)^. In recent years, it has been reported that social media use has significant effects on vaccine hesitancy and refusal^(12,29)^.

Participants view information dissemination through healthcare workers as a fundamental strategy. The literature indicates that healthcare professionals’ recommendations are decisive in vaccine acceptance^(21)^. Additionally, it has been reported that multisectoral interventions (health–media–education) are more effective in reducing misconceptions^(30)^.

The fact that public health messaging regarding herd immunity is often given lower priority suggests that this concept alone may not always serve as a strong motivator. The literature indicates that emphasizing herd immunity can have varying effects on individual motivation^(31)^. Therefore, it is considered necessary to address both individual benefits and social responsibility in a balanced manner in vaccination communication.

Overall, the findings highlight the importance of a multi- level intervention approach. Addressing media, health system, and community-based education components together may provide a more effective framework for combating vaccine hesitancy and refusal.

## LIMITATIONS

This study has several limitations. First, the study was conducted with midwives working at family health centers in the province of Uşak, Türkiye; therefore, the generalizability of the results to different regions or different groups of health professionals may be limited.

Since the data were collected based on self-reports, participant responses may have been influenced by recall bias. Additionally, although the data collection tool was developed based on the literature, it is not a standardized measurement instrument; this constitutes a limitation in terms of measurement validity.

The cross-sectional design of the study does not allow for the establishment of causal relationships between variables. Therefore, the findings should be interpreted within the framework of the participants’ views and observations.

## CONCLUSION

According to midwives’ reports, the most commonly cited factors contributing to vaccine hesitancy and refusal include exposure to anti-vaccine content online, concerns about vaccine ingredients, religious beliefs, past vaccination experiences, and perceptions of trust in the healthcare system.

The approaches recommended by midwives include media-based information dissemination, educational activities conducted by healthcare workers, community-based awareness campaigns, and regular vaccination campaigns.

In conclusion, this study highlights the current state of vaccine hesitancy and refusal based on the observations of midwives working in primary care. The findings are expected to contribute to future research in this field and the development of intervention strategies.

## DATA AVAILABILITY

The entire dataset supporting the results of this study is available upon request to the corresponding author.
