# Supplementary material for: Vaccine hesitancy and refusal: perspectives of midwives working in family health centers – a descriptive cross-sectional study
Source: Rev Esc Enferm USP. 2026 Sep 7;60:e20250469. doi: 10.1590/1980-220X-REEUSP-2025-0469en (PMC13577236; doi:10.1590/1980-220X-REEUSP-2025-0469en)
Supplement: Translation

# Translation: Reticencia y rechazo a la vacunación: perspectivas de parteras que trabajan en centros de salud – un estudio descriptivo transversal

**DOI:** 10.1590/1980-220X-REEUSP-2025-0469es

**Authors:** Şeyma Çatalgöl, Eda Can

**Affiliations:** 1 Uşak University, Faculty of Health Sciences, Department of Nursing, Uşak, Türkiye.; 2 İzmir City Hospital, İzmir, Türkiye.

**Keywords:** Partería, Vacunación, Negativa a la Vacunación, Vacilación a la Vacunación

## Abstract

**Objetivo:**

El presente estudio tiene como objetivo determinar las opiniones de las matronas que trabajan en centros de salud familiar sobre la reticencia y el rechazo a la vacunación, así como las estrategias de solución que proponen para abordar este problema.

**Método:**

Este estudio descriptivo transversal se llevó a cabo con 81 matronas que trabajan en centros de salud familiar de la provincia de Uşak, Turquía, entre el 24 de marzo y el 18 de mayo de 2021. Los datos se recopilaron a través de Google Forms utilizando un cuestionario estructurado elaborado a partir de la bibliografía. Los datos se analizaron utilizando estadísticas descriptivas, incluyendo frecuencias y porcentajes.

**Resultados:**

Según los informes de las matronas los factores más citados que contribuyen a la reticencia y al rechazo a la vacunación incluyen la exposición a sitios web antivacunas (90,1%), la preocupación por los ingredientes de las vacunas (70,4%), la influencia atribuida a los líderes comunitarios (66,7%) y las creencias religiosas (65,4%). Otros factores señalados incluyen experiencias previas con la vacunación (56,8%), la desconfianza hacia los fabricantes de vacunas (44,4%) y la desconfianza hacia los profesionales sanitarios y el sistema de salud (38,3%). Un análisis de las fuentes de información sobre vacunas reveló que los pacientes recurrían con mayor frecuencia a Internet (37,0%), sus círculos sociales (30,9%), las instituciones sanitarias (24,7%) y la televisión (7,4%). El 65,4% de las matronas informó de haber tenido contacto con personas antivacunas. Las estrategias recomendadas con mayor frecuencia incluyen: presentar contenidos televisivos que apoyen la vacunación (70,4%), emitir anuncios de servicio público a través de la televisión e Internet (67,9%) y proporcionar información sobre las vacunas a través de los profesionales sanitarios (58,0%).

**Conclusión:**

Según las observaciones de campo de las matronas, la reticencia y el rechazo a la vacunación se consideran un fenómeno multidimensional que implica una combinación de desinformación digital, problemas relacionados con la confianza y factores socioculturales. El refuerzo de las iniciativas educativas lideradas por los profesionales sanitarios y las estrategias de información a través de los medios de comunicación podrían contribuir a aumentar la aceptación de las vacunas.

## INTRODUCCIÓN

La reticencia y el rechazo a la vacunación se definen como el hecho de que las personas retrasen o rechacen la vacunación a pesar de la disponibilidad de los servicios de vacunación. Aunque la vacunación es una de las intervenciones de salud pública más rentables para prevenir enfermedades infecciosas, la reticencia y el rechazo a la vacunación siguen siendo un problema de salud mundial significativo en la actualidad. La Organización Mundial de la Salud (OMS) identificó la reticencia a la vacunación como una de las diez principales amenazas para la salud mundial en 2019^(1)^. Según los datos actuales, aproximadamente 14,3 millones de niños en todo el mundo seguían sin estar vacunados en 2024; a estos niños se les denomina “niños de dosis cero”. Durante el mismo período, se informó que la tasa de cobertura de vacunación mundial para la tercera dosis de la vacuna DTP3, que brinda protección contra la difteria, el tétanos y la tos ferina, era del 85%. Estos resultados indican que aún existen brechas significativas en los servicios de vacunación^(2)^.

La reticencia a la vacunación se define como un fenómeno asociado a numerosos factores, tales como preocupaciones sobre la seguridad de las vacunas, dificultades para acceder a ellas y negligencia individual. En este proceso, los trabajadores de la salud son considerados la fuente de información más confiable que puede influir en las decisiones de las personas con respecto a la vacunación y desempeñan un papel fundamental en la prestación de asesoramiento eficaz^(3)^. La capacidad de los trabajadores de la salud para generar confianza, aclarar la información contradictoria y ofrecer recomendaciones basadas en la evidencia se encuentra entre los factores clave para reducir la reticencia y el rechazo a la vacunación^(4,5)^.

Entre los trabajadores de la salud, las parteras ocupan una posición única y fundamental, especialmente en la atención primaria, debido al contacto continuo y cercano que establecen con las mujeres a lo largo del embarazo, el parto y el posparto. Las parteras asumen la responsabilidad de proporcionar información y orientación sobre las vacunas tanto maternas como infantiles. Dado el creciente énfasis en la vacunación durante el embarazo y la necesidad de que los padres estén informados sobre las vacunas durante el período prenatal, las parteras desempeñan un papel clave en el asesoramiento sobre vacunación y la prestación de servicios de vacunación^(6)^.

Manejar las conversaciones con personas que expresan reticencia o rechazo a las vacunas, abordar sus inquietudes y proporcionar información confiable se encuentran entre las responsabilidades profesionales y éticas fundamentales de las parteras. El cumplimiento de estas responsabilidades mediante un enfoque no crítico, respetuoso y centrado en la mujer permite a las parteras realizar contribuciones significativas a los procesos destinados a aumentar la aceptación de las vacunas y apoyar la salud pública^(7)^.

Sin embargo, los propios niveles de confianza de las parteras en las vacunas y sus posibles dudas pueden influir en su tendencia a recomendar vacunas a las personas. Se ha demostrado una fuerte relación entre la confianza de los trabajadores de la salud en las vacunas y la actitud del público hacia la vacunación^(8)^. Además, se ha informado de que las parteras pueden expresar mayores preocupaciones con respecto a la seguridad de las vacunas en comparación con otros trabajadores de la salud y pueden mostrarse más reacias a recomendar vacunas, particularmente durante el embarazo. Esto subraya la importancia de fortalecer los conocimientos y la confianza de las parteras con respecto a las vacunas^(9)^.

Por otro lado, el uso generalizado de las plataformas digitales influye significativamente en el flujo de información sobre las vacunas. Cuando los trabajadores de la salud, en particular las parteras, comparten información inexacta o antivacunas en entornos digitales, esto puede socavar la confianza del público en los mensajes de salud. Por lo tanto, proporcionar información científica y confiable tanto en la práctica clínica como en las plataformas digitales es una parte integral de las responsabilidades profesionales de las parteras^(10)^.

A pesar del papel fundamental que desempeñan las parteras en los servicios de vacunación, existe un número limitado de estudios que aborden de manera integral las experiencias clínicas de la vida real de las parteras, en particular las que trabajan en la atención primaria, en relación con la reticencia y el rechazo a la vacunación, así como las estrategias de solución que han desarrollado para abordar esta situación. La investigación actual se centra principalmente en intervenciones específicas, y las evaluaciones integrales basadas en las prácticas de campo siguen siendo insuficientes^(7,11)^.

En este contexto, los estudios que reflejan las prácticas de campo y se basan en las experiencias de las parteras son importantes para contribuir al desarrollo de enfoques que aborden la reticencia a la vacunación. En consecuencia, este estudio tiene como objetivo identificar las opiniones de las parteras que trabajan en centros de salud familiar con respecto a la reticencia y el rechazo a la vacunación, así como las estrategias de solución que proponen para esta situación.

## MÉTODOS

### Diseño del Estudio

Este estudio se diseñó como un estudio transversal descriptivo. Debido a su naturaleza descriptiva, no se realizaron análisis de relaciones o causalidad entre variables; los datos se evaluaron utilizando únicamente estadísticas descriptivas.

### Entorno del Estudio

La investigación se llevó a cabo en centros de salud familiar de la provincia de Uşak, ubicada en la región del Egeo Interior de Turquía. En Turquía, los servicios de atención primaria de salud se prestan en gran medida a través de los centros de salud familiar, y las parteras desempeñan un papel activo en los servicios de salud preventiva, como la atención prenatal, la atención posparto, la administración de vacunas infantiles y los servicios rutinarios de salud familiar. Por lo tanto, las parteras son profesionales de la salud clave que se enfrentan directamente a la reticencia y el rechazo a la vacunación.

Los centros de salud familiar donde trabajan las participantes cuentan con una plantilla de entre dos y seis médicos y parteras. Cada médico trabaja con una partera, y cada equipo atiende a una población de aproximadamente 4000 personas. Los programas de vacunación en Turquía se llevan a cabo de manera coordinada; mientras que la vacuna posnatal contra la hepatitis B se administra a los recién nacidos en el hospital, otras vacunas infantiles, las vacunas contra el tétanos para mujeres embarazadas y otras vacunas de rutina se administran en los centros de salud familiar. Este contexto proporciona un entorno de trabajo que podría influir directamente en las observaciones de las parteras con respecto a la reticencia y el rechazo a la vacunación.

### Criterios de Inclusión y Exclusión

El estudio incluyó a todas las parteras de entre 18 y 65 años que trabajaban en centros de salud familiar de la provincia donde se llevó a cabo la investigación y que dieron su consentimiento informado por vía electrónica. Las parteras que no dieron su consentimiento electrónico fueron excluidas del estudio.

### Participantes y Muestreo

El estudio se llevó a cabo entre el 24 de marzo y el 18 de mayo de 2021. La población del estudio consistió en un total de 88 parteras que trabajaban en 23 centros de salud familiar de la provincia en cuestión. Dado el tamaño relativamente pequeño y manejable de la población, se pretendía llegar a toda la población. Este enfoque tenía como objetivo minimizar el error de muestreo y mejorar la representatividad de los datos para las parteras que trabajan en centros de salud familiar en toda la provincia. De las 88 parteras que conformaban la población del estudio, 81 participaron voluntariamente en el estudio; siete parteras decidieron no participar. La tasa de participación voluntaria fue del 92%, lo que mejoró la representatividad de los datos. También se enviaron correos electrónicos de recordatorio para fomentar la participación.

### Herramienta de Recopilación de Datos

Los datos se recopilaron mediante un cuestionario elaborado de acuerdo con la literatura^(4,5,6,7,8,9,10,11,12,13)^, que constaba de 27 preguntas de opción múltiple. La encuesta constaba de cuatro secciones: (1) características descriptivas de las parteras; (2) percepciones sobre el rechazo o la reticencia a la vacunación; (3) percepciones sobre la reticencia y el rechazo a la vacunación, incluyendo creencias, fuentes de información y exposición profesional; y (4) soluciones propuestas para prevenir la reticencia o el rechazo a la vacunación.

### Validez y Fiabilidad

La validez de contenido del cuestionario fue evaluada por tres académicos especializados en partería. Se pidió a los expertos que revisaran cada ítem basándose en los criterios de cobertura (el grado en que el ítem abarca el tema relevante), claridad (si la redacción es clara y comprensible) y adecuación (la alineación del ítem con el objetivo de la investigación). A partir del feedback de los expertos, se realizaron cambios en la redacción y revisiones lingüísticas de algunos ítems.

Se llevó a cabo una prueba piloto de la encuesta con 15 parteras que trabajaban en una provincia diferente, lo que dio lugar a correcciones menores en la redacción y la elección de palabras en tres ítems; no se requirieron cambios en los ítems restantes. Las parteras que participaron en la prueba piloto no se incluyeron en el estudio principal.

Dado que el cuestionario constaba de múltiples secciones temáticas en lugar de una escala unidimensional, se calculó un coeficiente alfa de Cronbach global como indicador general de la consistencia interna (α = 0,87).

### Recolección de Datos

Debido a la pandemia de COVID-19 y a la necesidad de limitar el contacto presencial, los datos se recolectaron en línea. El cuestionario se preparó utilizando Google Forms y se distribuyó a las participantes por correo electrónico. Se enviaron mensajes de recordatorio para aumentar la participación.

### Aprobación Ética y Declaraciones de Consentimiento Informado

El estudio fue aprobado por el Comité de Ética en Investigación Clínica de la Universidad de Uşak (Fecha de aprobación: 17 de marzo de 2021, Número: 69-69-06) y se llevó a cabo de conformidad con las directrices éticas establecidas en la Declaración de Helsinki. También se obtuvo el permiso de la Dirección Provincial de Salud de Uşak, a la que están afiliados los servicios de atención primaria de salud involucrados en el estudio (Fecha: 3 de marzo de 2021, Número: E-49998565-604.02.03). Se obtuvo el consentimiento informado por escrito de las parteras que aceptaron participar en el estudio. Se informó a todas las participantes sobre el propósito y el diseño del estudio.

### Análisis Estadístico

Los datos se analizaron utilizando el paquete de software SPSS 22.0. Las variables categóricas se presentaron como frecuencia (n) y porcentaje (%). Debido a la naturaleza descriptiva del estudio, no se realizó ningún análisis de relación estadística o causalidad entre las variables.

## RESULTADOS

### Resultados Relativos a las Características Demográficas de las Parteras

Un total de 81 parteras participaron en el estudio. Las características sociodemográficas de las participantes se presentan en la Tabla 1. Se determinó que el 44,4% de las participantes se encontraba en el grupo de edad de 35 a 44 años y que el 85,2% estaba casada. En cuanto a la experiencia profesional, la duración más común fue de 6 a 10 años (28,4%) y de 0 a 5 años (24,7%). Se observó que la mayoría de las participantes tenía un (34,5%) o dos (38,3%) hijos (Tabla 1).

**Tabla 1:** Características descriptivas de las parteras (N = 81) – Uşak, TR, Türkiye, 2021.

	n = 81	(%)
**Edad**		
18–24	12	14,8
25–34	26	32,1
35–44	36	44,4
45–64	7	8,6
**Estado civil**		
Casado	69	85,2
Soltero	12	14,8
**Experiencia profesional (años)**		
0–5	20	24,7
6–10	23	28,4
11–15	11	13,6
16–20	11	13,6
21–25	10	12,3
26–30	4	4,9
≥31	2	2,5
**Número de hijos**		
0	10	12,3
1	28	34,5
2	31	38,3
3	11	13,6
≥4	1	1,2

### Informes de las Parteras Sobre la Reticencia y el Rechazo a la Vacunación

En la Tabla 2 se presentan los informes de las parteras sobre la reticencia y el rechazo a la vacunación. Entre los factores más frecuentemente señalados se encuentran la exposición a sitios web antivacunas (90,1%), la preocupación por los ingredientes de las vacunas (70,4%), la influencia atribuida a los líderes comunitarios (66,7%) y las creencias religiosas (65,4%). A estos les siguen las experiencias de vacunación anteriores (56,8%), la desconfianza hacia los fabricantes de vacunas (44,4%) y la desconfianza hacia los profesionales sanitarios y el sistema sanitario (38,3%) (Tabla 2).

**Tabla 2:** Percepciones de las parteras sobre el rechazo o la reticencia a la vacunación (N = 81) – Uşak, TR, Türkiye, 2021.

	Sí n (%)	No n (%)	Parcialmente n (%)
Creencias religiosas	53 (65,4)	10 (12,3)	18 (22,2)
Sitios web antivacunas	73 (90,1)	3 (3,7)	5 (6,2)
La influencia atribuida a los líderes comunitarios	54 (66,7)	10 (12,3)	17 (21)
Problemas de transporte a los centros de salud	22 (27,2)	42 (51,9)	17 (21)
Experiencias previas con la vacunación	46 (56,8)	20 (24,7)	15 (18,5)
Desconfianza hacia el personal sanitario y el sistema sanitario	31 (38,3)	26 (32,1)	24 (29,6)
Desconfianza hacia los fabricantes de vacunas	36 (44,4)	20 (24,7)	25 (30,9)
Preocupación por los ingredientes de las vacunas	57 (70,4)	16 (19,8)	8 (9,9)

La Tabla 3 presenta las percepciones de las parteras con res­pecto a las creencias individuales, las fuentes de información de las pacientes y la experiencia profesional en el contexto de la reticencia o el rechazo a la vacunación. Entre las preocupaciones más frecuentes en relación con las creencias individuales se encontraban el temor a que las vacunas pudieran tener efectos desconocidos a largo plazo (48,1%), que pudieran causar autismo o enfermedades autoinmunes (37,0%) y que no tuvieran beneficios (32,1%). A estas les seguían la percepción de que los fabricantes de vacunas son de origen extranjero (24,7%), la creencia de que los alimentos ecológicos pueden sustituir a las vacunas (22,2%) y que las vacunas pueden causar infertilidad (6,2%) (Tabla 3).

**Tabla 3:** Percepciones de las parteras sobre la reticencia y el rechazo a la vacunación: creencias, fuentes de información y exposición profesional (N = 81) – Uşak, TR, Türkiye, 2021.

Ítem	n (%)
Creencia de que los alimentos ecológicos sustituyen a las vacunas	18 (22,2)
Creencia de que las vacunas no tienen ningún beneficio	26 (32,1)
Creencia de que las vacunas causan autismo o enfermedades autoinmunes	30 (37)
Creencia de que las vacunas pueden tener efectos a largo plazo desconocidos	39 (48,1)
Origen extranjero de los fabricantes de vacunas	20 (24,7)
Creencia de que las vacunas pueden causar infertilidad	5 (6,2)
Percepciones de las parteras sobre las fuentes de información de las pacientes sobre las vacunas*	
Televisión	6 (7,4)
Internet	30 (37)
Entorno social (amigos/vecinos)	25 (30,9)
Centros sanitarios	20 (24,7)
**Exposición a casos de reticencia o rechazo a la vacunación**	
Encontrados	53 (65,4)
No se han encontrado	28 (34,6)

*Las categorías marcadas como “Se admiten respuestas múltiples” permiten a los encuestados seleccionar más de una opción. Los porcentajes se han redondeado a un decimal.

En cuanto a las fuentes de información, se determinó que los pacientes utilizan con mayor frecuencia Internet (37,0%), seguido del entorno social (30,9%), las instituciones sanitarias (24,7%) y la televisión (7,4%). El 65,4% de las parteras informó de haber tratado con personas antivacunas (Tabla 3).

### Soluciones Sugeridas para la Reticencia o el Rechazo a la Vacunación Entre las Parteras

En la Tabla 4 se presentan sugerencias para reducir la reticencia y el rechazo a la vacunación entre las parteras. Al revisar los resultados, es evidente que las sugerencias se centran en gran medida en campañas de información en los medios de comunicación y en actividades educativas llevadas a cabo a través de los profesionales sanitarios. El enfoque más recomendado fue incluir contenidos a favor de la vacunación en los programas de televisión (70,4%). La siguiente recomendación más frecuente fue la emisión regular de anuncios de servicio público en televisión e Internet (67,9%) y la educación pública a través de los profesionales sanitarios sobre los ingredientes de las vacunas, los efectos secundarios y los riesgos de no vacunarse (58,0%). Otras estrategias recomendadas incluían proporcionar información sobre los beneficios de las vacunas durante actividades de la sociedad civil (55,6%), campañas de vacunación periódicas (49,4%), educar a la población sobre la importancia de la vacunación en clases prenatales, asociaciones de mujeres y centros de educación pública (43,2%), información sobre la inmunidad colectiva por parte de profesionales sanitarios (40,7%) y políticas de vacunación obligatoria (40,7%) (Tabla 4).

**Tabla 4:** Soluciones propuestas por las parteras para prevenir la reticencia o el rechazo a la vacunación (N = 81) – Uşak, TR, Türkiye, 2021.

Soluciones propuestas por las parteras	n	%
Incluir contenido a favor de la vacunación en los programas de televisión	57	70,4
Proporcionar información sobre los beneficios de las vacunas durante actividades de la sociedad civil	45	55,6
Anuncios de servicio público en televisión e Internet	55	67,9
Campañas de vacunación periódicas	40	49,4
Educación pública a cargo del personal sanitario sobre los componentes de las vacunas, los efectos secundarios y los riesgos de no vacunarse	47	58
Educar a la población sobre la importancia de la vacunación en clases prenatales, asociaciones de mujeres y centros de educación pública	35	43,2
Información sobre la inmunidad colectiva por parte de los profesionales sanitarios	33	40,7
Política de vacunación obligatoria	33	40,7

## DISCUSIÓN

Este estudio demuestra que la reticencia y el rechazo a la vacunación presentan una estructura multidimensional, según las observaciones de campo realizadas por las parteras. Los resultados sugieren que las actitudes hacia la vacunación no se limitan únicamente a los niveles de conocimiento individuales, sino que surgen dentro de un marco complejo que debe considerarse en conjunto con los entornos de información digital, los sistemas de creencias culturales, los espacios de interacción social y las dinámicas de confianza institucional.

Las declaraciones de las participantes revelan que los entornos digitales, y en particular el contenido antivacunas, están adquiriendo una influencia cada vez mayor en los procesos de comunicación en materia de salud. El uso generalizado de plataformas en línea para acceder a información de salud puede facilitar la difusión de contenido no verificado a audiencias más amplias^(12)^. Restringir el contenido antivacunas en las redes sociales y otras plataformas digitales puede considerarse un paso importante para fortalecer la confianza en las vacunas^(13)^.

Las preocupaciones sobre los componentes de las vacunas y las percepciones de seguridad constituyen otro tema destacado en las opiniones de los participantes. La literatura indica que preocupaciones similares están muy extendidas entre diferentes poblaciones y que los conceptos erróneos sobre los efectos a largo plazo de las vacunas son particularmente influyentes^(14)^. Los hallazgos son consistentes con la literatura existente^(5,15)^, lo que indica que la percepción del riesgo y los problemas de confianza se encuentran en el centro de la reticencia a la vacunación.

Se estima que las estrategias de comunicación transparentes y basadas en la evidencia podrían desempeñar un papel fundamental en la reducción de estas percepciones^(16)^. Los factores sociales y culturales, en particular las creencias religiosas y la influencia de los líderes comunitarios, constituyen otro aspecto notable en las declaraciones de los participantes. La literatura también indica que las creencias religiosas pueden ser un factor determinante en los procesos de toma de decisiones sobre la vacunación^(17)^. Este hallazgo demuestra que los comportamientos de salud están determinados no solo por los procesos de toma de decisiones individuales, sino también por las normas culturales y religiosas.

Por lo tanto, se cree que las campañas de concientización llevadas a cabo a través de las autoridades religiosas podrían ayudar a reducir las brechas de información^(18)^. Las experiencias individuales y las experiencias negativas relacionadas con procesos de vacunación anteriores también surgen como un tema significativo. La literatura indica que las experiencias previas de efectos secundarios están asociadas con la reticencia a la vacunación^(19)^. Fortalecer la seguridad de las vacunas y monitorear de manera transparente los efectos secundarios podría ayudar a aliviar estas preocupaciones^(20)^.

La desconfianza hacia los fabricantes de vacunas es otro elemento clave destacado en las declaraciones de los participantes. La literatura indica que la percepción de las personas de que los fabricantes de vacunas se mueven por el lucro puede aumentar la reticencia a la vacunación^(14)^. Esto sugiere que aumentar la transparencia en los procesos de desarrollo y autorización de las vacunas podría ser crucial para generar confianza^(3)^.

La confianza en los trabajadores de la salud y en el sistema de salud es otro tema clave que surgió en las opiniones de los participantes. La literatura indica que las habilidades de comunicación de los profesionales de la salud y su capacidad para construir relaciones basadas en la confianza desempeñan un papel fundamental en el aumento de la aceptación de las vacunas^(21)^. Este hallazgo destaca la necesidad de fortalecer no solo la transmisión de información, sino también la comunicación basada en la confianza en las intervenciones.

Las observaciones de campo de las parteras apuntan a la diversidad de las fuentes de información de las personas. Se ha informado que Internet, los círculos sociales, las instituciones de salud y los medios tradicionales son fuentes de información efectivas en diversos grados. La literatura enfatiza que los entornos de información digital forman un ecosistema complejo que da forma a las decisiones de salud^(22)^. Por lo tanto, es crucial fortalecer la posición de los profesionales de la salud como fuentes confiables de información.

Según los participantes, los factores clave que contribuyen a la reticencia y el rechazo a las vacunas incluyen preocupaciones sobre los efectos a largo plazo, asociaciones con el autismo y las enfermedades autoinmunes, una percepción de ineficacia y la desconfianza hacia los fabricantes. Además, se han reportado conceptos erróneos como la creencia de que los alimentos orgánicos son protectores, así como que las vacunas conllevan el riesgo de infertilidad. La literatura indica que dicha desinformación se difunde particularmente a través de plataformas digitales y erosiona la confianza en las vacunas^(23,24,25)^. Por lo tanto, mejorar la alfabetización en salud y desarrollar estrategias de comunicación específicas revisten gran importancia^(26)^.

En el estudio, una proporción significativa de parteras informó haber encontrado casos de reticencia y rechazo a las vacunas. Este hallazgo es consistente con la literatura que indica que la reticencia y el rechazo a las vacunas son prevalentes en diversas poblaciones^(27,28)^. Esta situación subraya la importancia de mejorar la alfabetización en salud y difundir información confiable a través de los profesionales de la salud.

Entre las recomendaciones de las parteras, destacan las campañas de concientización en los medios de comunicación y las estrategias de información impartidas por los trabajadores de la salud. En particular, los anuncios de servicio público que se transmitirán por televisión e Internet se consideran un área clave de intervención. Este hallazgo concuerda con estudios que resaltan la influencia de los medios de comunicación en la aceptación de las vacunas^(29)^.

En los últimos años, se ha informado de que el uso de las redes sociales tiene efectos significativos en la reticencia y el rechazo a la vacunación^(12,29)^. Los participantes consideran que la difusión de información a través de los trabajadores de la salud es una estrategia fundamental. La literatura indica que las recomendaciones de los profesionales de la salud son decisivas para la aceptación de las vacunas^(21)^. Además, se ha informado de que las intervenciones multisectoriales (salud-medios de comunicación-educación) son más eficaces para reducir los conceptos erróneos^(30)^.

El hecho de que a menudo se dé menor prioridad a los mensajes de salud pública sobre la inmunidad colectiva sugiere que este concepto por sí solo puede no servir siempre como un fuerte motivador. La literatura indica que enfatizar la inmunidad colectiva puede tener efectos variables en la motivación individual^(31)^. Por lo tanto, se considera necesario abordar tanto los beneficios individuales como la responsabilidad social de manera equilibrada en la comunicación sobre la vacunación.

En general, los hallazgos resaltan la importancia de un enfoque de intervención multinivel. Abordar conjuntamente los componentes de los medios de comunicación, el sistema de salud y la educación comunitaria puede proporcionar un marco más eficaz para combatir la reticencia y el rechazo a la vacunación.

## LIMITACIONES

Este estudio presenta varias limitaciones. En primer lugar, el estudio se llevó a cabo con parteras que trabajan en centros de salud familiar en la provincia de Uşak, Turquía; por lo tanto, la generalización de los resultados a diferentes regiones o a diferentes grupos de profesionales de la salud puede ser limitada.

Dado que los datos se recolectaron a partir de autoinformes, las respuestas de las participantes pueden haber estado influidas por un sesgo de recuerdo. Además, aunque la herramienta para la recolección de datos se desarrolló basándose en la literatura, no es un instrumento de medición estandarizado; esto constituye una limitación en términos de validez de la medición.

El diseño transversal del estudio no permite establecer relaciones causales entre las variables. Por lo tanto, los resultados deben interpretarse dentro del marco de las opiniones y observaciones de las participantes.

## CONCLUSIÓN

Según los informes de las parteras, los factores más comúnmente citados que contribuyen a la reticencia y el rechazo a la vacunación incluyen la exposición a contenido antivacunas en línea, las preocupaciones sobre los ingredientes de las vacunas, las creencias religiosas, las experiencias de vacunación pasadas y las percepciones de confianza en el sistema de salud.

Los enfoques recomendados por las parteras incluyen la difusión de información a través de los medios de comunicación, actividades educativas llevadas a cabo por trabajadores de la salud, campañas de concientización comunitarias y campañas de vacunación periódicas.

En conclusión, este estudio destaca el estado actual de la reticencia y el rechazo a la vacunación basándose en las observaciones de las parteras que trabajan en la atención primaria. Se espera que los hallazgos contribuyan a futuras investigaciones en este campo y al desarrollo de estrategias de intervención.

## DISPONIBILIDAD DE DATOS

El conjunto completo de datos que respalda los resultados de este estudio está disponible previa solicitud al autor correspondiente.
